# Enhancement of Polypropylene Bonding Through Plasma–Ultrasonic Treatment

**DOI:** 10.3390/polym17060726

**Published:** 2025-03-10

**Authors:** Hui Wang, Chuhao Yang, Limei He, Binbin Yu, Xiaobin Zhao, Zongbin Huang

**Affiliations:** 1 Hubei Key Laboratory of Advanced Technology for Automotive Components, Wuhan University of Technology, Wuhan 430070, China; huiwang@whut.edu.cn (H.W.); ych1049732204558@whut.edu.cn (C.Y.); 2 Hubei Longzhong Laboratory, Xiangyang 441000, China; 3 Hubei Collaborative Innovation Center for Automotive Components Technology, Wuhan 430070, China; 4 Guangxi Laboratory of New Energy Automobile, SAIC GM Wuling Automobile Co., Ltd., Liuzhou 545001, China; binbin.yu@sgmw.com.cn (B.Y.); xiaobin.zhao@sgmw.com.cn (X.Z.); zongbin.huang@sgmw.com.cn (Z.H.)

**Keywords:** polypropylene bonding, plasma–ultrasonic treatment, molecular dynamics

## Abstract

In response to the issue of the insufficient adhesion strength of polypropylene materials, a plasma–ultrasonic treatment is proposed. Plasma treatment is first conducted to activate the polypropylene adherends, and then ultrasonic vibration is applied to the adhesive to facilitate the interface contact, enhancing the bonding performance of polypropylene. The shear strength of the test specimens was assessed using single-lap shear tests. The bonding samples were characterized by scanning electron microscopy (SEM) and energy-dispersive X-ray spectroscopy (EDS), contact angle, and infrared analysis to explore the bonding mechanism of plasma–ultrasonic treatment. The results show that compared to untreated polypropylene specimens, the plasma treatment process increased the shear strength of the polypropylene specimens by 370.3%, and the addition of ultrasonic-assisted technology further increased the shear strength of the polypropylene specimens by 10.6%. The coefficient of variation decreased from 0.53 in the untreated sample to 0.32 for the plasma–ultrasonic treatment, enhancing the stability of adhesion. Plasma treatment introduces active groups, such as hydroxyl groups, onto the surface of polypropylene and increases the surface roughness of polypropylene. Ultrasonic treatment promotes the penetration of adhesive microstructures on the surface of polypropylene, enhancing the anchoring effect of the adhesive, thereby improving bonding performance. Furthermore, through molecular dynamics analysis, compared to the untreated polypropylene bonding system, the bonding energy of the bonding system under the plasma–ultrasonic treatment was increased by 57%, effectively enhancing the shear strength of polypropylene bonding. Plasma–ultrasonic treatment can effectively improve the bonding strength of polypropylene, providing a new idea for the study of polymer bonding.

## 1. Introduction

Polypropylene (PP) is a thermoplastic resin made from the polymerization of propylene monomers and characterized by excellent chemical resistance, heat resistance, high strength, and toughness [1,2]. In the automotive field, PP is commonly used for door trim, bumpers, side panels, and other components due to its durability, wear resistance, and ease of maintenance [3].

With the development needs of automotive lightweighting, plastic tailgates use PP materials for both inner and outer panels and are bonded together through adhesive processes. Due to the challenges in controlling the energy during welding, the poor joint strength, and the fact that welding can cause some degree of damage to the components, the automotive tailgate is typically joined using adhesive bonding. However, the low surface energy, high chemical inertness, low surface roughness, and the presence of weak boundary layers of PP collectively lead to difficulties in bonding and insufficient strength [4]. Solving these problems typically requires special adhesives and surface treatment technologies to improve the bonding performance of PP plastics.

Currently, the physical methods for modification mainly include sanding and sandblasting [5,6]. Chemical methods [7] include surface treatments such as plasma treatment [8,9], coupling agent treatment [10], and anodization [11]. Mandolfino et al. [12] demonstrated the improvement of adhesion performance of polyethylene treated with plasma in terms of wettability, evaluated by contact angle measurement and the lap shear strength of adhesive joints achieved using treated surfaces. Mandolfino et al. [13] also used plasma to treat the polypropylene surface, and the results showed that plasma treatment increased the surface roughness of polypropylene, and more oxygen-containing polar groups were introduced into the treated polypropylene surface, which enhanced the hydrophilicity and chemical activity of the surface and helped to improve the bonding performance. Sanchis et al. [14] treated polyethylene film with plasma, greatly increasing the surface energy value of polar components, indicating surface activation due to the generation of polar groups (mainly =CO, -COOH, and -OH groups) and also revealed a slight increase in surface roughness due to surface etching. Hao Du et al. [15] studied the interaction between O or OH radicals and the PP surface. The high reactivity of the O radical and the high efficiency of hydrogen atom extraction make it show higher efficiency in the surface modification, which is helpful to further improve the surface modification effect of polypropylene. The plasma treatment process increased the surface roughness of PP and changed the chemical properties of the PP surface. However, due to the viscosity of the adhesive itself, the liquid adhesive could not well fill the surface microstructure through the penetration action, resulting in poor interface anchoring and severely limiting the adhesion strength.

Ultrasonic vibration-assisted bonding processes have a wide range of research applications. Wang et al. [16] applied ultrasonic vibration to the adhesive joints of carbon fiber-reinforced polymer (CFRP) and aluminum alloys, and the surfaces of the adhesive joints were treated with sandpaper grinding, resulting in an adhesive interface with interlocking anchors. Du et al. [17] used ultrasonic vibration to treat SU-8 photoresist molds, effectively improving the interfacial bonding strength. The cavitation rate of the SU-8 film treated with ultrasonic vibration was 34.4% higher than that of the SU-8 film without ultrasonic treatment. Holtmannspotter et al. [18] employed ultrasonic cavitation to eliminate impurities from the adherend, thereby reducing their impact on bonding strength. Sundukov [19] demonstrated that the cavitation of ultrasonic waves leads to the destruction of polymer chains, reducing the viscosity of adhesives and thereby improving their fluidity and wettability, and can enhance the ability of the adhesive to fill irregular structures at the micron and submicron levels on the bonding surface, thereby increasing bonding strength. Avraham et al. [20] studied the ultrasonic welding characteristics of PEEK (polyether ether ketone) and graphite fiber reinforced composite (APC-2), and showed that the ultrasonic welding parameters had a significant effect on the welding effect of PEEK composite. By optimizing welding parameters, high strength welded joints can be obtained while avoiding material degradation. These studies all indicate that the application of ultrasonic waves can promote various joining processes and enhance the strength of adhesion. In previous work, no one had applied ultrasonic technology to PP bonding. PP is an inert material, and it is difficult to bond. Ultrasonic treatment can change the contact status and improve its wettability. Good wettability helps the adhesive spread on the PP surface to form a closer contact.

This article employs the method of orthogonal experiments to analyze the impact of a plasma–ultrasonic treatment. The bonding strength of the samples is assessed through shear tests, and scanning electron microscopy (SEM) is used to analyze the morphology and elemental changes of the treated PP surface. Infrared spectroscopy technology is also utilized to analyze the functional groups on the PP surface. Finally, molecular dynamics simulation is applied to simulate the process of ultrasonic-assisted bonding under plasma treatment conditions to explore the impact of plasma–ultrasonic-assisted bonding processes on PP.

## 2. Materials and Methods

### 2.1. Materials

In this work, the substrate used is a PP (SCG Chemicals, Bangkok, Thailand) plate. Table 1 lists the main mechanical properties.

A two-component acrylic adhesive (provided by Plexus, Danvers, CO, USA) is used to bond the joints. The A component of acrylic adhesive is composed of methyl methacrylate, and the B component is composed of benzoyl peroxide. At room temperature, the main component of the adhesive and the curing agent are mixed in a mass ratio of 10:1. Table 2 lists some of its main technical characteristics. The acrylic adhesive is a polar adhesive; the existence of polar molecules gives it good adhesion properties. The polar adhesive can not only enhance the bonding strength but also improves the stability of the bond, ensuring more durable and reliable connections. Since the surface of PP is non-polar, it is necessary to treat the surface of PP to achieve a good bonding effect.

### 2.2. Surface Pre-Treatment Process

**Figure 1:**
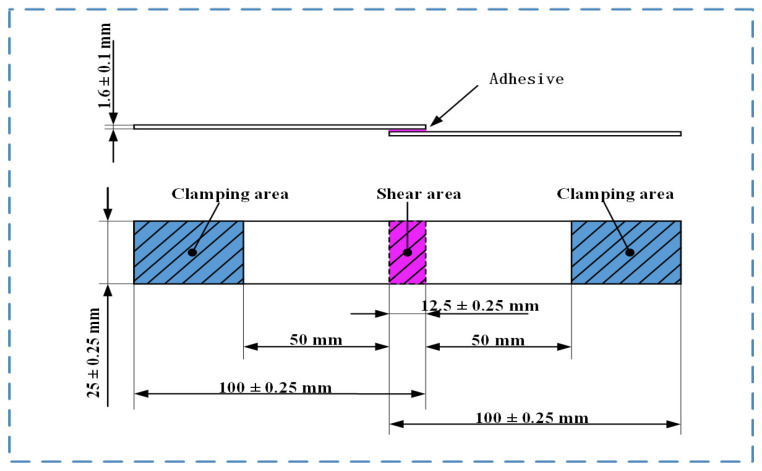
Bonded joint shapes and size.

According to ASTM D3136, the dimensions of the PP plate (length × width × height) are 101.6 mm × 25.4 mm × 2 mm, the bonding area (length × width) is 25 mm × 12.5 mm, and the thickness of the adhesive layer is 0.2 mm. After the bonding is completed, the excess adhesive at the edge is removed and allowed to cool naturally at room temperature. To ensure the accuracy of the test, the tensile shear strength is tested after 24 h. The shape and size of the bond joint are shown in Figure 1.

**Figure 2:**
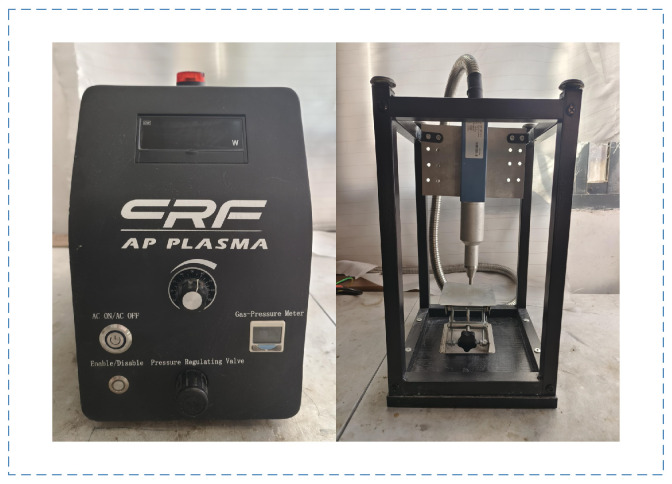
The plasma treatment equipment.

**Figure 3:**
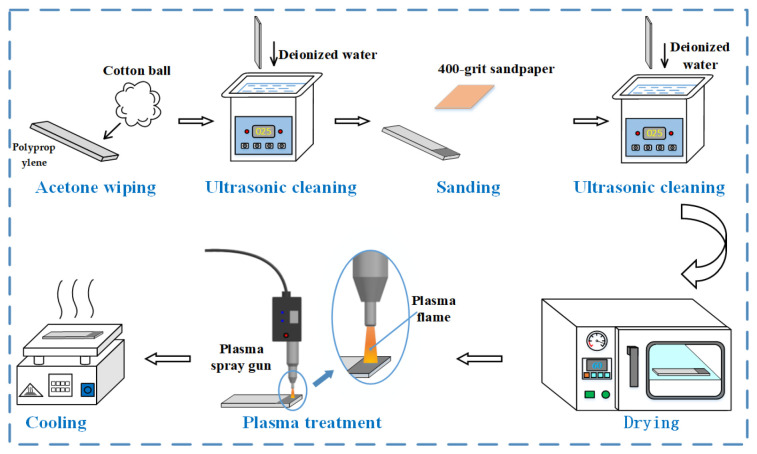
Plasma treatment process for PP.

The surface of the PP plate is wiped with acetone to remove surface oils and other contamination. Then, the PP plate is placed in deionized water for ultrasonic cleaning for 5 min to remove large particles from the surface. The bonding surface of the PP plate is sanded with 400-grit sandpaper, placed in deionized water for ultrasonic cleaning, and then dried in a 60 °C drying oven for 5 min. The surface of the PP plate to be bonded is treated with a plasma gun and allowed to cool naturally to room temperature before bonding. The plasma treatment equipment is shown in Figure 2. By use of the power knob, the power of the plasma treatment is adjusted. The PP plate is placed on the platform, and the treatment distance is ensured by adjusting the knob. The whole plasma treatment process is carried out at room temperature. The plasma treatment process is shown in Figure 3.

The parameters that significantly affect the plasma modification effect include the treatment distance, treatment time, and treatment power. According to the instructions of the plasma machine, discharge instability occurs when the treatment power is below 100 W. The actual treatment power generally ranges from 100 W to 300 W, so the treatment power is chosen at three levels: 100 W, 200 W, and 300 W. The typical treatment distance for the plasma treatment machine is 5–20 mm, so three distance levels are selected: 5 mm, 10 mm, and 15 mm. Based on preliminary exploratory experiments, the treatment time is initially determined to be between 20 and 80 s, and the time is chosen at three levels: 30 s, 50 s, and 70 s. The orthogonal factors of plasma treatment are shown in Table 3.

**Figure 4:**
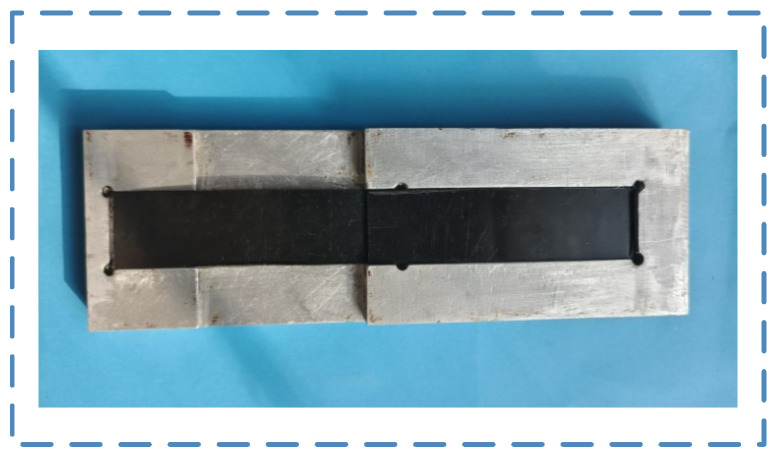
Bonding fixture.

In order to ensure the quality of the bonded joint, 7075 aluminum alloy is used to manufacture the fixture used for bonding. The bonding fixture is shown in Figure 4. The fixture is designed as two cavities to house the polypropylene used for bonding, with a height difference of 1.7 mm. Through the position of the fixture cavity, the area of the bonding area can be controlled at 25 mm × 12.5 mm, and it is used to ensure the thickness of the adhesive layer. After the PP is treated, the adhesive is applied to the bonding surface of two pieces of PP, which takes 2 min to complete. Two pieces of PP applied with adhesive were overlapped on the fixture and cured for two hours at room temperature and pressure after removing excess adhesive.

### 2.3. Ultrasonic Treatment Process

**Figure 5:**
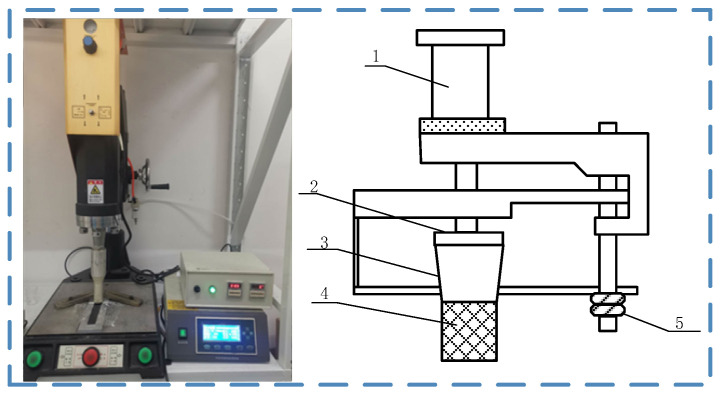
Experimental platform of ultrasonic treatment: (1) hydraulic equipment; (2) transducer; (3) amplitude converter; (4) ultrasonic vibration head; (5) fastening bolt.

Figure 5 shows the ultrasonic vibration equipment, ME-1800, produced by Maxwide Ultrasonic Co., Ltd., Shanghai, China. The equipment is composed of an ultrasonic generator, a transducer, an amplitude converter, an ultrasonic vibration head, and a hydraulic equipment. The ultrasonic generator converts the electrical signals into high-frequency electrical signals, and the transducer receives these electrical signals and converts them into high-frequency mechanical vibrations. During the experiment, the fixture equipped with the adhesive joint is placed on the ultrasonic experimental platform. The amplitude and vibration time of the ultrasonic vibration are set through the control box, and the gap height is controlled by the fastening bolt.

**Figure 6:**
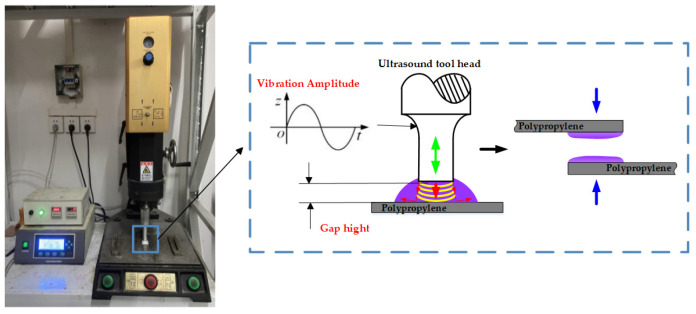
Ultrasonic treatment.

For the pre-treated plates, ultrasonic treatment is applied to the bonding area of the PP plate after glue application. The ultrasonic treatment introduces ultrasonic vibration into the adhesive. The prepared adhesive is applied to the bonding surface of the PP, and the sonotrode is controlled to be submerged in the adhesive with a certain gap left to the PP surface, thereby applying ultrasonic vibration to the adhesive on the surface. The ultrasonic treatment process is shown in Figure 6.

Because of the curing behavior, the adhesive begins to cure gradually with the increase of vibration time, which impairs the effect of ultrasonic treatment and thus affects the bonding strength. In addition, if the ultrasonic vibration amplitude is too low, it cannot promote the full anchorage of the adhesive and the substrate; if the ultrasonic amplitude is too large, it will generate a large amount of heat in a short time, accelerate the curing of the adhesive, and decrease the strength of the bond. Based on previous testing experience, gap height, vibration time, and vibration amplitude are chosen as the three factors for the orthogonal experiment, and their levels are shown in Table 4.

### 2.4. Characterization

#### 2.4.1. Shear Strength Test

The tensile shear strength of PP single-lap joints is tested using a universal tensile testing machine (SANS CMT5205 manufactured by MTS Systems (China) Co., Ltd., Beijing, China). The experiment is conducted at room temperature with a speed of 2 mm/min. Five samples are tested for each group, and the average value is calculated. The shear strength is determined by Equation (1).

(1) $$\tau =\frac{F}{B\times L}$$

In the formula, τ represents the shear strength (MPa), *F* represents the maximum shear force (N), and *B* and *L* represent the width and length of the bonding area (mm).

#### 2.4.2. Contact Angle Measurement

The basic principle of contact angle measurement is based on Young’s equation, which describes the equilibrium relationship between a droplet on a solid surface and the solid surface itself. The size of the contact angle can reflect the wettability of a liquid on a solid surface. When the contact angle is less than 90°, the surface exhibits hydrophilicity; when the contact angle is greater than 90°, the surface exhibits hydrophobicity. Contact angle tests were conducted on PP surfaces that were untreated, sanded, and plasma-treated, respectively, to determine the impact of different treatment processes on the PP surface.

#### 2.4.3. Surface Morphology and Chemical Composition Analysis

**Figure 7:**
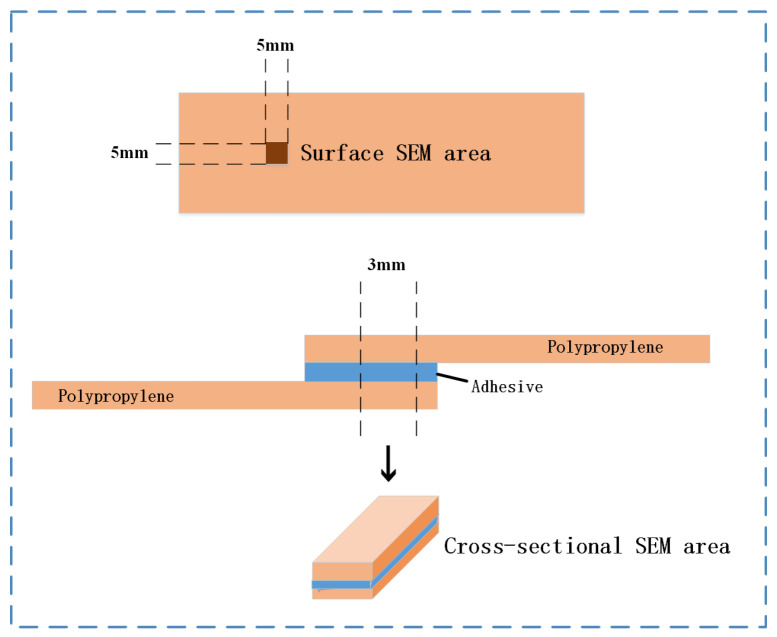
Surface morphology and cross-sectional morphology observation.

Observation and analysis of PP surfaces and joint morphologies under different treatment processes are conducted using a scanning electron microscope (SEM). Energy-dispersive X-ray spectroscopy (EDS) is utilized to analyze the elemental composition of the surfaces. The SEM equipment used is the Zeiss field emission scanning electron microscope, Gemini SEM 300 (Carl Zeiss Microscopy GmbH, Oberkochen, Germany), which is equipped with an EDS spectrometer. The magnification is 10–1,000,000, with automatic calibration according to the acceleration voltage and the distance. To observe the surface morphology of PP, a 5 mm × 5 mm slice is cut using water cutting. To observe the cross-sectional morphology of the PP bonding sample, a 3 mm wide slice is cut from the center of the bonding area, as shown in Figure 7.

Atomic force microscope (AFM) tests are carried out on the PP surfaces to obtain two-dimensional and three-dimensional topography and surface roughness values, which are used to analyze the differences in surface morphology and surface roughness of PP under different surface treatments.

#### 2.4.4. Surface Contact Angle and Chemical Functional Group

To analyze the differences in surface wettability of PP under different treatments, the contact angle of the surface of PP is measured. The functional groups on the surface of PP are detected by FTIR tests to analyze the influence of surface functional groups with different surface treatments.

### 2.5. Molecular Dynamics Simulation

**Figure 8:**
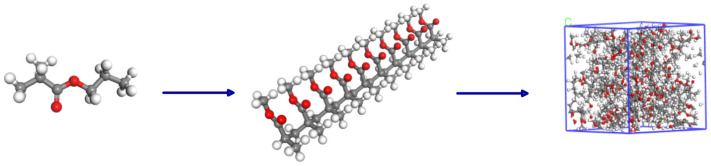
Adhesive model construction.

In this work, the degree of polymerization of methyl methacrylate is set to be 1. A model is built based on the structural formula, and 10 methyl methacrylate monomer chains are placed in a periodic boundary box to construct an amorphous adhesive unit cell with a density of 1.16 g/cm^3^ and dimensions of 26.0 × 26.0 × 26.0 Å^3^, as shown in Figure 8. In the model, the red sphere represents the O atom, the gray sphere represents the C atom, and the white sphere represents the H atom. COMPASS force field is a general and suitable polymer force field, which can describe the molecular structure and dynamic behavior of PP. COMPASS force field is used to assign molecular parameters such as bonds and angles to methyl methacrylate. Through geometric optimization, the energy of the model is minimized, and a stable structure is obtained. After geometric optimization, the balance between the temperature and pressure of the system is achieved through NVT so that the temperature is maintained at 298 K, and the pressure standard is one atmosphere.

**Figure 9:**
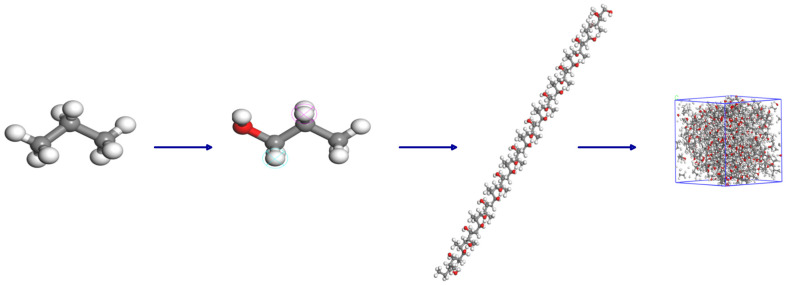
PP model construction.

Hydroxyl groups are introduced into the PP molecules, and a model is built based on this structural formula. The charge numbers are calculated by the Forcefield force field to assign the correct charges to the model. The molecules are used as the repeating unit, with a molecular chain length of 10, to construct a PP chain. Through the Amorphous Cell module, 10 PP chains are placed in a periodic boundary box using the Monte Carlo method, constructing a PP unit cell with dimensions of approximately 26.0 × 26.0 × 26.0 Å^3^. COMPASS force field is used to assign molecular parameters such as bonds and angles to PP. The energy of PP is optimized by geometric optimization. In the optimization process, the initial configuration of the molecule is ensured to avoid excessive geometric distortion. At the same time, dynamic relaxation is performed within the NVT ensemble to eliminate internal stress, with a relaxation temperature of 298 K and a relaxation time of 30 ps. The molecular dynamics model of PP is shown in Figure 9.

**Figure 10:**
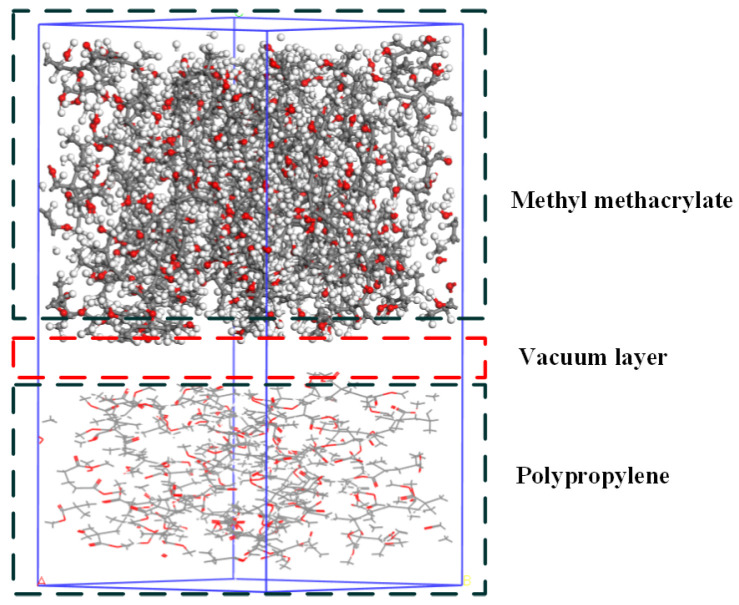
Relaxation optimized adhesive model.

The adhesive model is constructed by placing the adhesive model above the PP model using the Build Layer method to create a bonding model with dimensions of approximately 34.89 × 34.89 × 124.48 Å^3^. The model undergoes 500 steps of geometric optimization to obtain the adhesive microstructure filling model, as shown in Figure 10. By adding pressure above the bonding model and setting the frequency of ultrasonic vibration to 25 kHz and the amplitude to 24 µm, the ultrasonic treatment process is simulated. For easy distinction, the PP model is represented by a stick model. The velocity-verlet algorithm is suitable for most molecular dynamics simulations and can efficiently calculate the displacement and velocity of atoms. The dynamic behavior of PP molecules under simulated ultrasonic treatment is observed by using the velocity-Verlet algorithm for molecular dynamics simulation.

## 3. Results

### 3.1. Optimization of Plasma Treatment Parameters

Based on Table 3, the orthogonal experiment for plasma treatment was designed using Minitab software (V19), as shown in Table 5. According to this experimental design, plasma treatment experiments were conducted, with each scheme repeated five times, and then the average value of the bonding strength was taken as the bonding strength, as shown in Table 5. The analysis of the experimental results is shown in Table 6.

**Figure 11:**
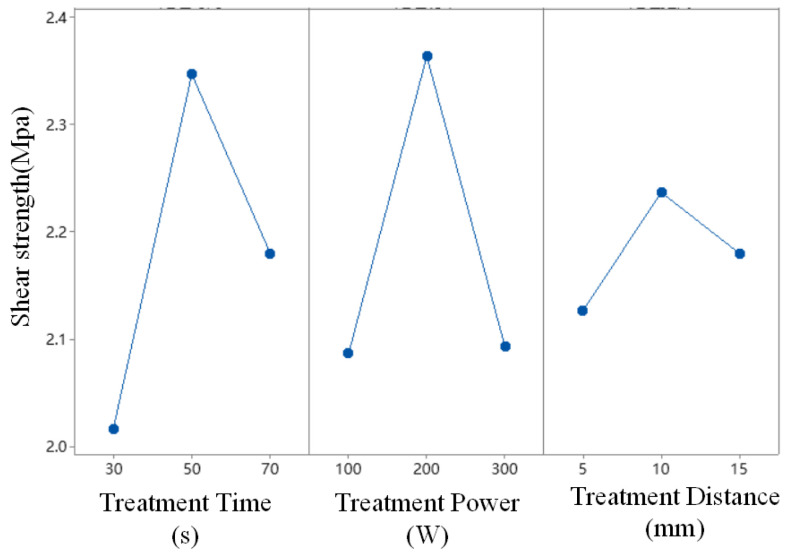
Main effect plot of the mean of the plasma treatment.

The data in Table 6 represent the average bonding strength obtained at different levels for each factor. The term “Delta” in the table indicates the difference between the maximum and minimum mean values obtained at different levels of that factor, reflecting the extent of the factor’s influence on the bonding strength. Based on the ranking order, the influence of the plasma parameters on the bonding strength in descending order is treatment time > treatment power > treatment distance. Figure 11 shows the trend of bonding strength variation with different levels of plasma treatment factors, from which the following optimal combination of plasma parameters can be derived: a treatment time of 50 s, a treatment power of 200 W, and a treatment distance of 10 mm. Since this optimal parameter combination is not included in the plasma treatment orthogonal experiment, an additional set of experiments is required for verification.

### 3.2. Ultrasonic Adhesive Application Optimization

After treating the PP surface with the optimal plasma treatment scheme, the adhesive is applied on the bonding surface. On this basis, the same plasma scheme is used to optimize the parameters of ultrasonic vibration. A total of 16 orthogonal test schemes are obtained, as shown in Table 7. The results of the tensile tests for each scheme are also recorded in Table 7. The test results are analyzed using Minitab software. The mean response is shown in Table 8, and the mean main effect curve is shown in Figure 8.

**Figure 12:**
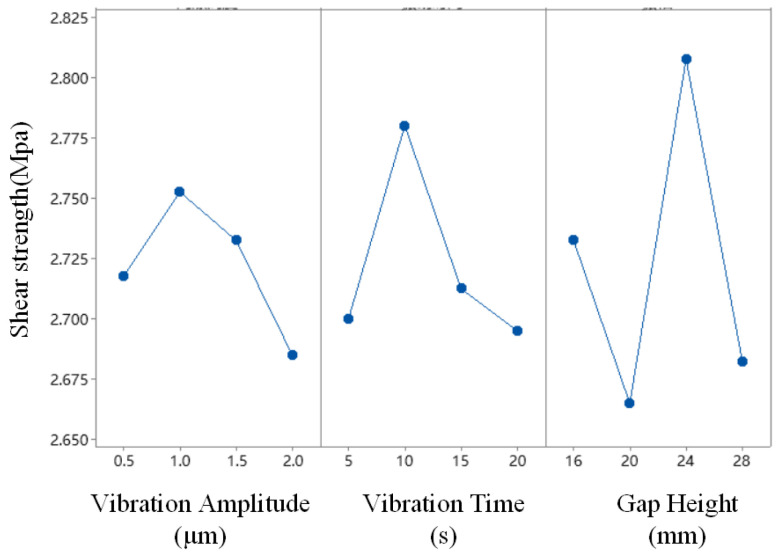
Ultrasonic treatment main effect plot of mean.

As shown in Figure 12, among the factors of vibration amplitude, vibration time, and gap height, the bonding strength increases with the values but decreases after the peaks. The data in Table 8 represent the mean bonding strength obtained at different levels for each factor. Based on the ranking order, the influence of ultrasonic parameters on bonding strength in descending order is vibration amplitude > vibration time > gap height. Figure 10 shows the trend of bonding strength variation with different levels of ultrasonic treatment factors, from which the following optimal parameters for the ultrasonic treatment can be derived: a gap height of 1.0 mm, a vibration time of 10 s, and a vibration amplitude of 24 μm. Since this optimal scheme is not included in the orthogonal experiment, an additional set of experiments is required for verification.

### 3.3. Shear Strength Testing Results

**Figure 13:**
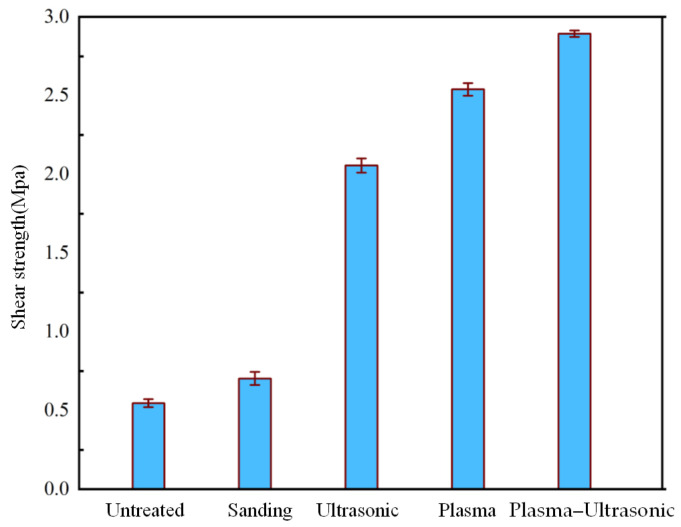
Shear strength testing results.

Shear tests were conducted using the bonding samples treated with the optimal plasma and ultrasonic parameters, as shown in Figure 13. The results of untreated joints and sanding-only ones are also shown in this figure for comparison. It can be observed that without any treatment, the bonding strength of PP is only 0.54 MPa (the 95% confidence interval for the mean difference was [0.52, 0.56] MPa), which increases to 0.69 MPa (the 95% confidence interval for the mean difference was [0.65, 0.73] MPa) after sanding treatment, a 9.5% increase compared to the untreated state. The bonding strength of the samples was increased to 2.05 MPa with the ultrasonic treatment (the 95% confidence interval for the mean difference was [1.99, 2.11] MPa). With the optimal plasma treatment, the bonding strength of the samples increases to 2.54 MPa (the 95% confidence interval for the mean difference was [2.51, 2.58] MPa), a 370.3% increase compared to the untreated state. The bonding strength of the sample was increased to 2.81 MPa by plasma–ultrasonic treatment (the 95% confidence interval for the mean difference was [2.79, 2.83] MPa), a further 10.6% increase compared to that with the plasma treatment alone. Meanwhile, plasma–ultrasonic treatment resulted in a change in the coefficient of variation from 0.73 in sanding treatment to 0.32. By comparing the enhancement effects of different surface modification processes on bonding strength, it can be concluded that the ultrasonic treatment after the plasma treatment of PP results in the best increase in shear strength of the bonding samples. The confidence intervals under different processing methods are shown in Table 9.

### 3.4. Surface Morphology and Chemical Composition Analysis

**Figure 14:**
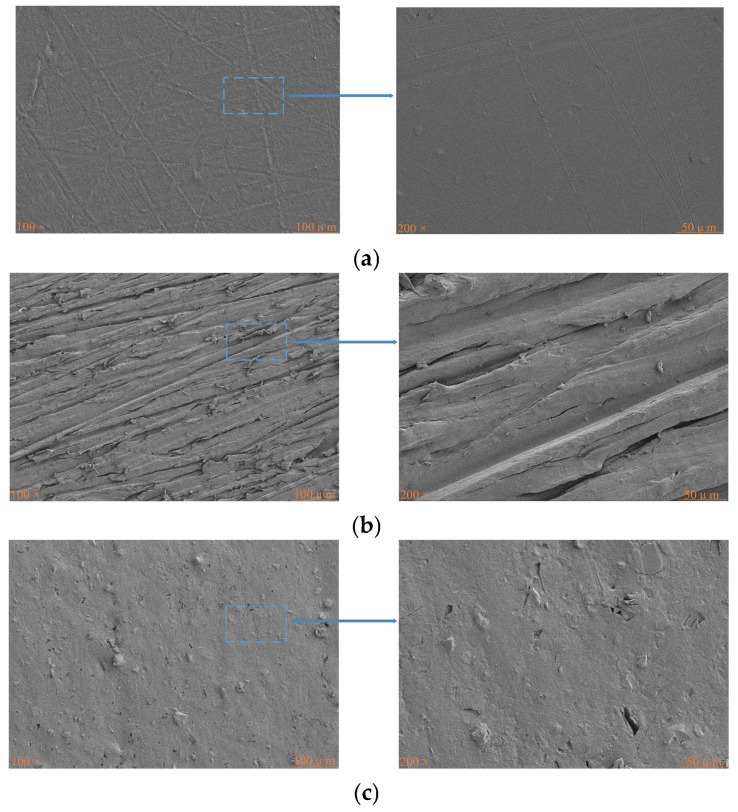
Surface morphology of PP under different treatments: (**a**) untreated, (**b**) sanding treatment, (**c**) plasma treatment.

The surface morphology of PP under different treatment processes is depicted in Figure 14. Figure 14a represents the surface of untreated PP, which has minor scratches formed during transportation; Figure 14b shows the surface of PP after the sanding treatment. The dense hydrophobic layer on the PP surface has been removed partially, increasing the surface roughness and providing certain conditions for mechanical interlocking. However, due to insufficient sanding, hydrophobic substances still remain on the surface. Figure 14c displays the surface of PP after the plasma treatment, where the dense hydrophobic layer on the PP surface has completely disappeared due to the high-temperature effect of the plasma treatment (through follow-up contact angle tests and infrared tests). In order to obtain accurate roughness values, atomic force microscope testing is conducted.

**Figure 15:**
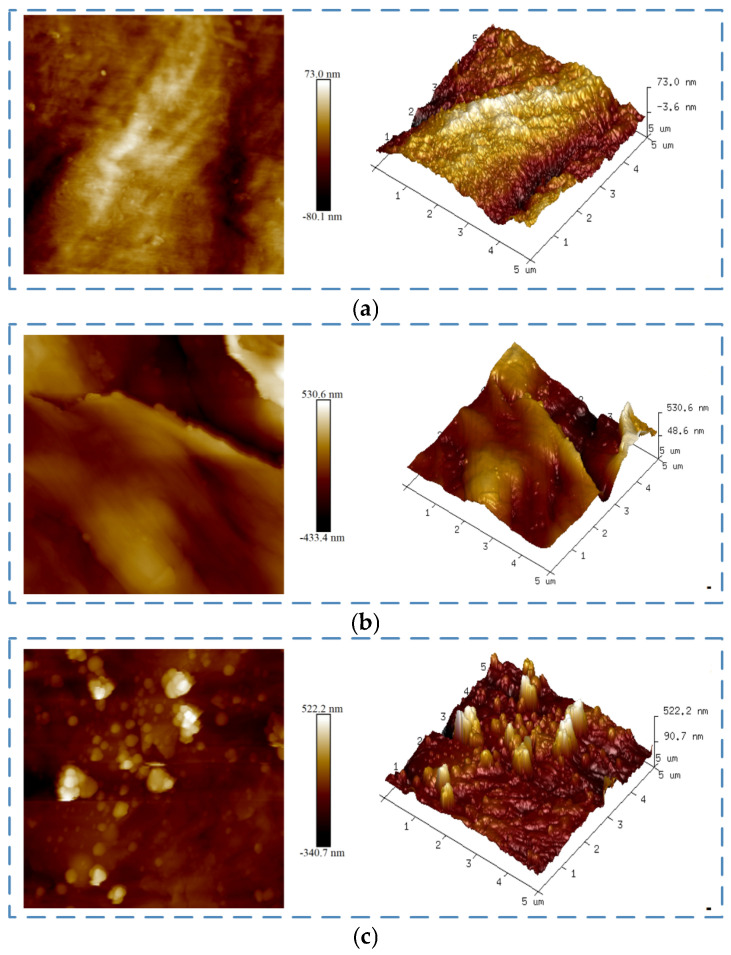
Atomic force microscope under different treatments: (**a**) untreated, (**b**) sanding treatment, (**c**) plasma treatment.

PP surfaces were scanned by atomic force microscope (AFM). Figure 15 shows the two–dimensional and three-dimensional topography of the surface, and the corresponding roughness values are shown in Table 10. Figure 15a represents the surface of untreated PP; the values of the surface *R_a_* and *R_q_* are 18.3 nm and 22.6 nm, respectively, and the height difference *R_max_* between the highest peak and the lowest valley is 144 nm. After the sanding treatment, the surface of PP is shown in Figure 15b; the values of its surface roughness *R_a_* and *R_q_* are 170 nm and 130 nm, which is about six times higher than that of the untreated PP, and the height difference *R_max_* between the highest peak and the lowest valley is also greatly improved, reaching 994 nm. Figure 15c shows the morphology of PP after the plasma treatment; the values of the surface *R_a_* and *R_q_* are 46.3 nm and 36.6 nm, respectively, and the height difference *R_max_* between the highest peak and the lowest valley is 417 nm. The increase in surface roughness enlarges the potential contact area between the adhesive and the surface of PP, which can improve the bonding strength. Although the surface roughness of PP after the plasma treatment is lower than that after the sanding treatment, the bonding strength is higher. It is necessary to analyze from the perspectives of surface wettability, surface elements, and surface functional groups to reveal the treatment mechanism.

**Figure 16:**
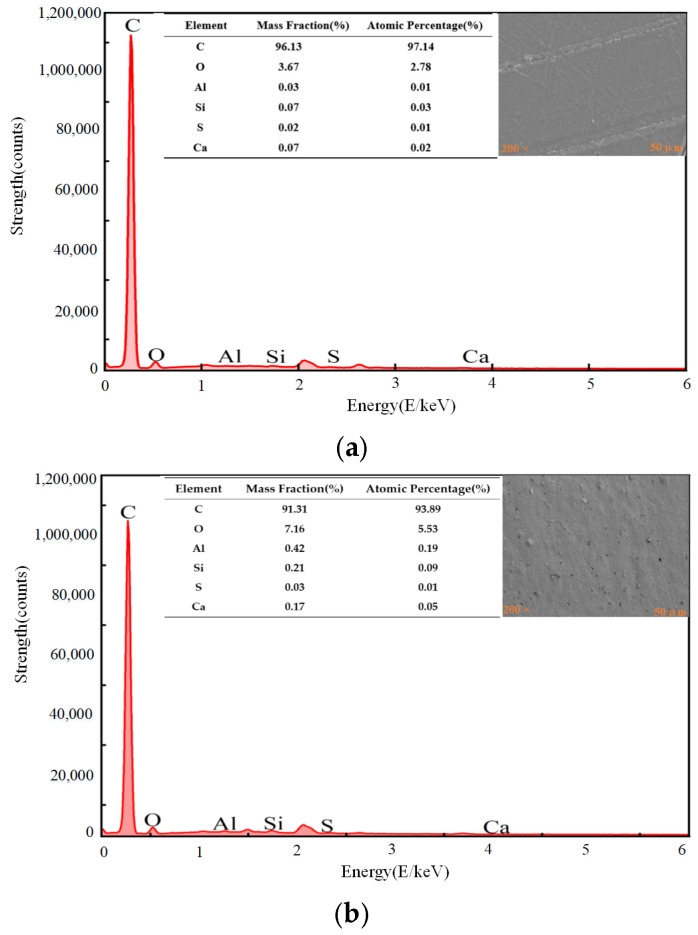
Atomic percentage under different treatments: (**a**) untreated, (**b**) plasma treatment.

As shown in Figure 16, the elemental composition of the untreated PP surface is as follows: the atomic percentage of C is 96.13%, and the atomic percentage of O is 3.67%. In addition, due to the presence of contaminants, there are also small amounts of elements such as Mg, S, Fe, etc. After the plasma surface treatment, the atomic percentages change obviously, with C decreasing to 91.31% and O increasing to 7.16%. Treating the PP surface with plasma technology can introduce oxygen-containing active groups, which significantly enhances the surface chemical activity, thereby improving the bonding performance of PP.

**Figure 17:**
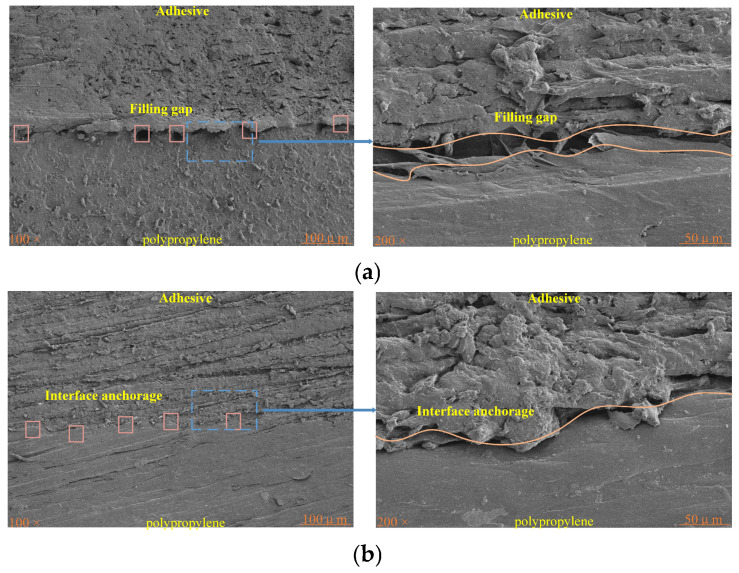
Cross-sectional view of the (**a**) traditional method and (**b**) plasma–ultrasonic method.

The AFM test results show that the surface roughness of plasma-treated PP is significantly higher than that of untreated PP but lower than that of sanded PP. The shear results (from Figure 11) show that the bonding strength obtained after the plasma treatment is higher than that after the sanding treatment. It can be seen that roughness does not determine bonding strength. According to mechanical anchoring theory, surface roughness is only one of the conditions to improve bonding strength, and surface roughness will increase the contact area between the adhesive and the interface, but penetration is also one of the key conditions. The ultrasonic treatment can reduce the viscosity of the adhesive owing to the thermal effect, making it easier to penetrate into the microgroove of the adhesive and at the same time improve the wettability of the adhesive to the surface of the adhesive (Figure 17), increase the contact area with the adhesive, and thus promote penetration. From the above analysis, it can be concluded that plasma treatment provides good surface roughness, and ultrasonic treatment makes the adhesive better contact with the interface to form mechanical anchorage, thereby improving the bonding effect.

**Figure 18:**
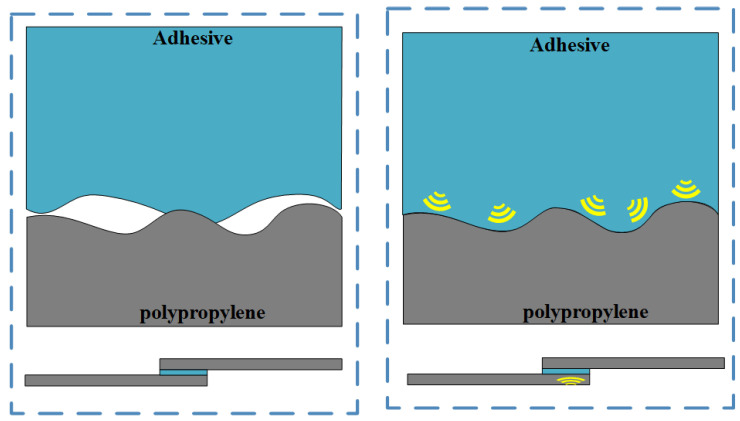
Schematic diagram of ultrasonic-facilitated filling.

Additionally, Figure 17 shows the cross-sectional view of the traditional method and ultrasonic method under different multiples. Under the traditional method, even though the PP interface has a high roughness that provides some mechanical interlocking conditions, there are many inadequately filled gaps between the adhesive layer and PP, and the existence of these gaps affects the bonding quality. Under the plasma–ultrasonic treatment, the filling gap disappears, and the interface anchorage is formed between the adhesive and PP, which greatly improves the bonding quality. The ultrasonic tool is constantly vibrating and impacting the adhesive layer, and the adhesive can fully penetrate and fill the surface of the adhesive plate. After curing, a mechanical interlock is formed between the adhesive and the PP plate, which strengthens the bonding joint. This also explains the reason for the maximum bonding strength under plasma–ultrasonic treatment in Figure 13. The ultrasonic action promotes the capillary action at the interface, which provides a driving force for the adhesive, reduces the viscosity of the adhesive, improves the fluidity of the adhesive, makes the adhesive fully fill the joint, forms the interface anchorage, and improves the bonding strength [21]. Figure 18 shows the schematic diagram. In Figure 17a, the pink boxes represent the fill gap. In Figure 17b, the pink box represents the filling mechanical anchorage.

### 3.5. Surface Contact Angle and Chemical Functional Group

**Figure 19:**
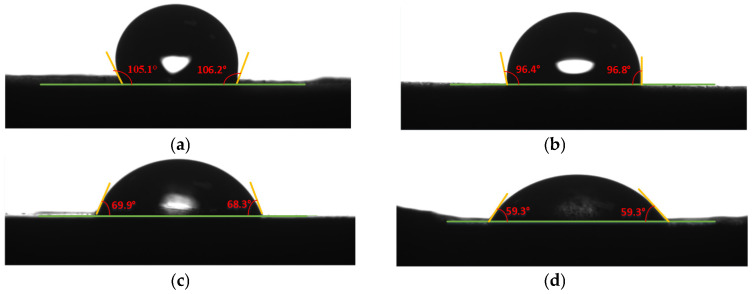
Contact angle of PP under different treatments: (**a**) untreated, (**b**) sanding treatment, (**c**) plasma treatment, (**d**) ultrasonic treatment.

Deionized water is used to conduct contact angle tests on the surfaces of PP plates treated with different surface modification processes at room temperature. The results are shown in Figure 19, and the corresponding contact angle values are shown in Table 11. The average of the left and right contact angles is taken as the final contact angle.

The contact angle on the untreated PP surface is 106.67°, which is similar with the angle of 103.67° reported by Chen et al. [22], and the contact angle is 97.68° after the sanding treatment. The difference is not significant, indicating that the sanding treatment has little effect on the wettability of the PP surface. After the plasma treatment, the contact angle becomes 70.01°, which is a significant change compared to the sanding treatment, indicating that the plasma treatment has a substantial impact on the wettability of the PP surface. Mandolfino et al. also confirmed that the contact angle becomes 68.31°after plasma treatment [20]. Morent et al. demonstrated that an increase in the polarity component of PP under plasma treatment leads to a decrease in the contact angle, and he has obtained similar effects on other polymers [23]. Additionally, after applying ultrasonic vibration to the plasma-treated PP surface, the contact angle measured is 58.96°. The results indicate that the plasma treatment significantly improves the surface activity of PP, and the wetting and spreading of deionized water under ultrasonic action are much better than those without ultrasonic action. The promotion of the ultrasonic action allows the adhesive to fully penetrate the substrate surface before its complete curing, enhancing the mechanical interlocking at the bonding interface and achieving higher bonding strength.

**Figure 20:**
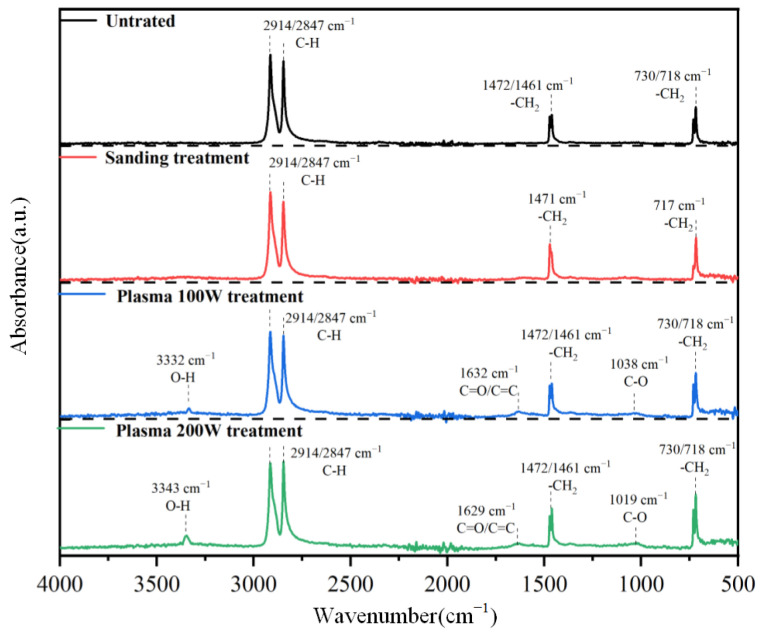
Infrared spectra of different treatment methods.

The infrared spectral characteristics of PP samples under different treatments are shown in Figure 20. The untreated PP sample mainly exhibits the typical infrared characteristics of PP, with absorption peaks at 2914 cm^−1^ and 2847 cm^−1^ attributed to the symmetric/asymmetric stretching vibration modes of the methyl C-H groups, absorption peaks at 1472 cm^−1^ and 1461 cm^−1^ attributed to the bending vibration modes of the methyl C-H groups in PP, and absorption peaks at 730 cm^−1^ and 718 cm^−1^ mainly attributed to the crystal vibration absorption bands of PP, related to the C-H vibration modes of unsaturated olefins. The PP sample treated by sanding also shows the typical characteristics of PP near 2914 cm^−1^, 2847 cm^−1^, 1471 cm^−1^, 1461 cm^−1^, 730 cm^−1^, and 718 cm^−1^, with no significant changes compared to the untreated PP sample. For PP samples treated at different powers of 100 W and 200 W, they also exhibit the typical characteristics of PP, but a new O-H stretching vibration absorption peak appears near 3340 cm^−1^, C=O and C=C stretching vibration characteristic peaks appear near 1630 cm^−1^, and a C-O stretching vibration absorption peak appears near 1038 cm^−1^. The relative intensity of each absorption peak increases with the plasma power. It is noted that the plasma treatment process of PP introduces hydroxyl groups. As a polar functional group, the carboxyl group can form hydrogen bonds or other chemical bonds with polar groups in the adhesive, facilitating chemical activity [24] and thereby enhancing the chemical bonding force between the adhesive and PP.

The shear test results in Figure 11 show that the bonding strength of the samples under ultrasonic treatment only reaches 2.05 MPa, which is higher than the 0.69 MPa obtained with the sanding treatment but lower than the 2.54 MPa obtained after the plasma treatment. Under ultrasonic treatment alone, the strength is improved mainly by enhancing mechanical interlocking, while under the plasma treatment, both chemical bonding and mechanical anchorage occur owing to the change of wettability and chemical functional group, which leads to higher bonding strength. However, after the plasma–ultrasonic treatment, the adhesive strength of the sample is further improved to 2.81 MPa, which can be attributed to the enhancement of mechanical interlocking by ultrasonic effect, for the ultrasonic had little influence on chemical bonding from the characterization. According to the above analysis, chemical bonding can effectively improve bonding strength, but in the whole plasma–ultrasonic treatment, mechanical interlock accounts for a larger proportion and has a greater impact on the bonding effect.

### 3.6. Molecular Dynamics Simulation Analysis

In the bonding process, the interaction between the adhesive molecule and the surface of the bonded material (such as van der Waals force, hydrogen bond, etc.) provides the bonding strength. This interaction can be seen as a kind of “microscopic binding energy”. Binding energy reflects the tightness of the combination of various components. The interfacial binding energy can be calculated from the difference in energy between the parts, as shown in Equation (2).

(2) $$E_{binding}=E_{pp}+E_{Adhesive}-E_{total}$$

In the equation, *E_pp_* represents the surface energy of PP, *E_Adhesive_* represents the molecular energy of the adhesive, and *E_total_* represents the total energy of the system. According to the analysis of the simulation results, the interfacial binding energies for the samples without plasma treatment, with plasma treatment, and with plasma–ultrasonic treatment are presented in Table 12. Based on Equation (2), it is indicated that the stronger the interfacial binding energy, the more stable the structure obtained. In the PP adhesive system with the plasma–ultrasonic treatment, the interfacial binding energy is 297.9 kcal/mol, which is a 9.4% increase compared to the adhesive system with only plasma treatment and a 57% increase compared to the adhesive system without plasma treatment. The increase of binding energy usually means a stronger interaction between atoms or molecules, which results in a more stable bond. That is, under the plasma–ultrasonic treatment, the interfacial binding energy is greater, leading to a more robust interfacial bond.

**Figure 21:**
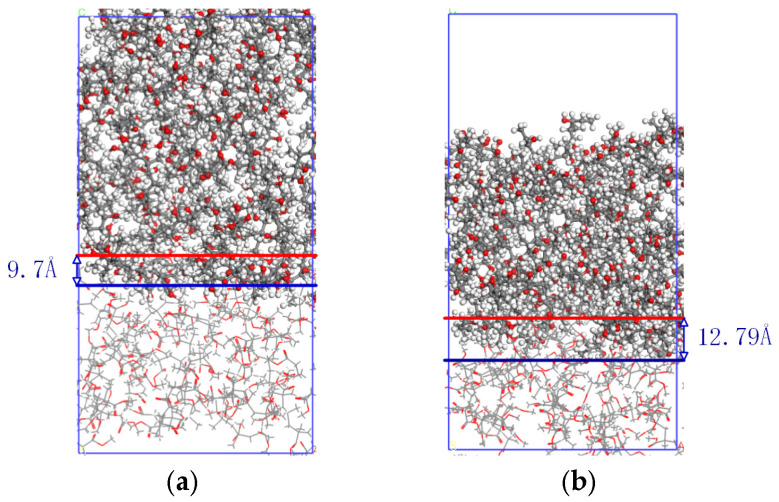
Interfacial microstructure filling results: (**a**) the micro-filling model of the adhesive without ultrasonic treatment, (**b**) the micro-filling model of the adhesive under the ultrasonic treatment.

To explore the impact of adhesive microstructure filling, a comparative analysis was conducted for 50 ps, yielding the interfacial microstructure filling results shown in Figure 21. Figure 21a represents the micro-filling model of the adhesive without ultrasonic treatment, with a filling depth of 9.7 Å; Figure 21b represents the micro-filling model of the adhesive under the ultrasonic treatment, with a filling depth of 12.79 Å. In terms of filling depth, the adhesive fills 31.8% deeper under ultrasonic treatment compared to that without ultrasonic treatment. This agrees well with the cross-sectional morphology shown in Figure 19. A greater filling depth indicates that more adhesive can enter the microstructure of the PP surface, forming better mechanical interlocking and thereby enhancing the bonding performance.

**Figure 22:**
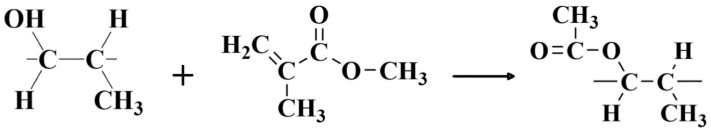
Chemical equation.

If hydroxyl groups are introduced on the PP surface with the plasma treatment, a reaction between hydroxylated PP and methyl methacrylate is allowed. In this reaction, the hydroxyl group of hydroxylated PP reacts with the carboxyl of methyl methacrylate to form an ester bond (-COO-). The chemical reaction equation is shown in Figure 22.

**Figure 23:**
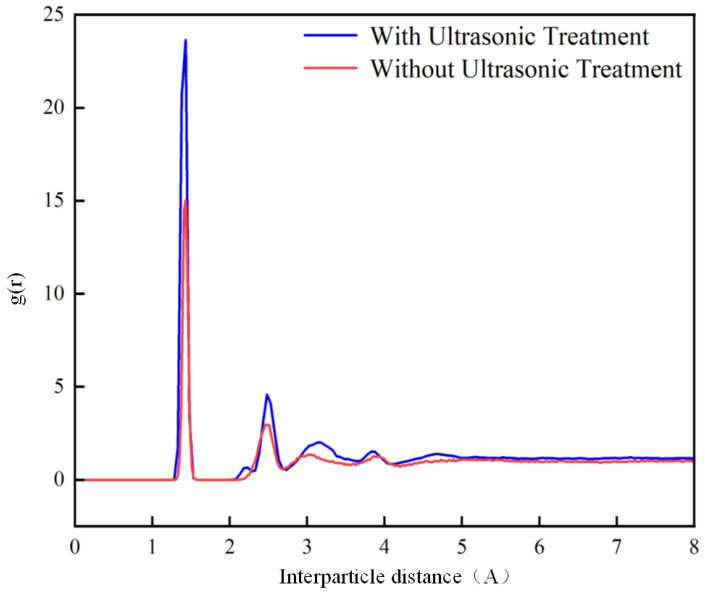
Radial distribution function.

To further analyze the mechanism by which ultrasonic treatment affects bonding, the radial distribution functions (RDFs) in the adhesive were calculated under conditions with and without ultrasonic treatment. The result is shown in Figure 21. The RDF is a statistical method used to describe the microstructure of a material, defined as the distance function of a specific particle to other particles around. The RDF peak at 1.43 Å is the characteristic peak of -COO-. Under the assistance of ultrasonic treatment, the number of -COO- bonds formed is much greater than that without ultrasonic action, indicating that the ultrasonic treatment causes more PP and methyl methacrylate to react, as shown in Figure 16. The ultrasonic action promotes reactions between the PP and the methyl methacrylate, forming more chemical bonds and thereby strengthening the interface bonding. Furthermore, between 2 Å and 3 Å, which is the peak range of hydrogen bonds, there are stronger peaks in the plasma–ultrasonic bonding compared to the plasma bonding. The increased hydrogen bond also strengthens the interface bonding. The RDF functions under different processing methods are shown in Figure 23.

Molecular dynamics simulations reveal several key atomic interactions that enhance bond energy observed in plasma–ultrasonic-treated PP systems. These interactions can be roughly classified as chemical bonding, hydrogen bonding, van der Waals forces, and changes in molecular conformation. The number of ester bonds increased by 60% compared to the untreated sample, directly contributing to the higher bond energy. The interfacial hydrogen bond density after plasma–ultrasonic treatment is significantly higher than that of untreated samples, which is closely related to the increase of interfacial bond energy.

## 4. Conclusions

To address the issue of low bonding strength in PP, a new plasma–ultrasonic treatment process is proposed. By studying the shear strength, microstructure, elemental changes, surface contact angle, and chemical functional group of samples prepared using different treatments, the following conclusions are drawn:

(1) By the optimum process parameters of plasma–ultrasonic treatment, the plasma treatment process increased the shear strength of the PP specimens by 370.3% compared with untreated specimens, and the addition of ultrasonic treatment further increased the shear strength of the PP specimens by 10.6%. The coefficient of variation decreased from 0.53 in the untreated specimens to 0.32 for the plasma–ultrasonic ones, enhancing the stability of adhesion.
(2) The plasma–ultrasonic treatment makes full use of the surface roughness and wettability provided by plasma treatment, and under the ultrasonic action, the adhesive can better penetrate into the PP surface. By integrating plasma treatment with ultrasonic treatment, the bonding strength is further improved.
(3) Molecular dynamics results show that the plasma–ultrasonic process increases the binding energy of the adhesive system by 57%. Additionally, the plasma– ultrasonic process results in more bonds within the adhesive system, leading to a tighter interface bonding.

The plasma–ultrasonic treatment has significant advantages in improving the bonding properties of PP materials. It can be widely used in the bonding of automotive interior parts (such as instrument panels, door panels, etc.). Although the plasma–ultrasonic treatment has shown significant advantages in industrial applications, there are still some potential limitations in real-world environments. Environmental humidity, temperature, and other conditions may affect the surface properties of PP and then affect the bonding effect. In production, environmental conditions need to be strictly controlled to ensure the stability of bonding quality. Future research and applications need to further optimize process parameters, reduce costs, and explore broader material compatibility to drive wider adoption of the technology.

## Figures and Tables

**Table 1:** PP properties.

Property	PP
Yield stress	29 MPa
Elongation at break	40%
Tensile modulus of elasticity	527 MPa

**Table 2:** Adhesive properties.

Property	Adhesive
Tensile strength	15.6 MPa
Elastic modulus	413.7 MPa
Elongation at break	45%

**Table 3:** Orthogonal factor levels under plasma treatment.

Levels	Factors		
	Treatment Power (W)	Treatment Time (s)	Treatment Distance (mm)
1	100	30	5
2	200	50	10
3	300	70	15

**Table 4:** Orthogonal factor levels under ultrasonic treatment.

Levels	Factors		
	Gap Height (mm)	Vibration Time (s)	Vibration Amplitude (µm)
1	0.5	5	16
2	1	10	20
3	1.5	15	24
4	2	20	28

**Table 5:** Plasma treatment orthogonal schemes and results.

Scheme	Treatment Time (s)	Treatment Power (W)	Treatment Distance (mm)	Average Shear Strength (MPa)
1	30	100	5	1.85
2	30	200	10	2.26
3	30	300	15	1.94
4	50	100	10	2.32
5	50	200	15	2.51
6	50	300	5	2.21
7	70	100	15	2.09
8	70	200	5	2.32
9	70	300	10	2.13

**Table 6:** Plasma treatment mean response (unit: MPa).

Levels	Treatment Time	Treatment Power	Treatment Distance
1	2.017	2.087	2.127
2	2.347	2.363	2.237
3	2.180	2.093	2.180
Delta	0.330	0.277	0.110
Rank	1	2	3

**Table 7:** Ultrasonic treatment orthogonal test scheme and results.

Scheme	Gap Height (mm)	Vibration Time (s)	Vibration Amplitude (µm)	Average Shear Strength (MPa)
1	0.5	5	16	2.66
2	0.5	10	20	2.75
3	0.5	15	24	2.71
4	0.5	20	28	2.75
5	1	5	16	2.63
6	1	10	28	2.88
7	1	15	24	2.73
8	1	20	24	2.77
9	1.5	5	28	2.94
10	1.5	10	16	2.68
11	1.5	15	20	2.72
12	1.5	20	28	2.59
13	2	5	24	2.57
14	2	10	20	2.81
15	2	15	20	2.69
16	2	20	16	2.67

**Table 8:** Ultrasonic treatment mean response (unit: MPa).

Levels	Gap Height	Vibration Time	Vibration Amplitude
1	2.718	2.700	2.732
2	2.752	2.780	2.665
3	2.732	2.712	2.808
4	2.685	2.695	2.683
Delta	0.067	0.085	0.143
Rank	3	2	1

**Table 9:** 95% confidence interval for results of shear strength testing (unit: MPa).

Group	Mean	95% Confidence Interval
Untreated	0.54	0.52–0.56
Sanding	0.69	0.65–0.73
Ultrasonic	2.05	1.99–2.11
Plasma	2.54	2.51–2.58
Plasma–Ultrasonic	2.81	2.79–2.83

**Table 10:** PP roughness under different treatment methods.

Treatment Method	*R_a_* (nm)	*R_q_* (nm)	*R_max_* (nm)
Untreated	18.3	22.6	144
Sanding Treatment	170	130	993
Plasma Treatment	46.3	36.6	417

**Table 11:** Contact angle of PP under different treatments.

Treatment Method	Left Contact Angle (°)	Right Contact Angle (°)	Average Contact Angle (°)
Untreated	105.1	106.2	105.6
Sanding Treatment	96.4	96.8	96.7
Plasma Treatment	69.9	68.3	69.1
Plasma–Ultrasonic Treatment	59.3	59.3	59.3

**Table 12:** Cohesive energy of adhesive systems under different treatments.

Treatment Method	PP Energy (kcal/mol)	Adhesive Energy (kcal/mol)	Total Energy (kcal/mol)	Interfacial Binding Energy (kcal/mol)
Untreated	−381.4	4959.9	4389	188.5
Plasma Treatment	−462.1	4876.2	4145	269.1
Plasma–Ultrasonic Treatment	−462.1	4979.1	4250	297.9

## Data Availability

The original contributions presented in this study are included in the article. Further inquiries can be directed to the corresponding author(s).
